# Pyelonephritis and Bacteremia Caused by *Klebsiella variicola* following Renal Transplantation

**DOI:** 10.1155/2021/9988396

**Published:** 2021-09-22

**Authors:** Cody Lo, Shazia Masud, Gregory D. Deans

**Affiliations:** ^1^Department of Ophthalmology, Faculty of Medicine, University of Ottawa, Ottawa, Ontario, Canada; ^2^Fraser Health, Surrey, British Columbia, Canada; ^3^Department of Pathology and Laboratory Medicine, Faculty of Medicine, University of British Columbia, Vancouver, British Columbia, Canada; ^4^Division of Infectious Diseases, Department of Medicine, Faculty of Medicine, University of British Columbia, Vancouver, British Columbia, Canada

## Abstract

*Klebsiella variicola* (*K. variicola*) is a Gram-negative organism genetically similar to *Klebsiella pneumoniae* (*K. pneumoniae*) that can cause a variety of diseases in humans. Bacteremia due to *K. variicola* is associated with a higher mortality rate than bacteremia with *K. pneumoniae*. Here, we describe a 65-year-old woman who developed pyelonephritis 2 months after receiving a renal transplantation following a longstanding history of end-stage renal disease secondary to polycystic kidney disease. Her creatinine on admission was unchanged from her posttransplant baseline, and an abdominal CT scan showed inflammatory changes around the transplanted kidney that were suggestive of an infection rather than allograft rejection. She was initially treated empirically with meropenem given a history of extended-spectrum beta-lactamase- (ESBL-) producing *E. coli* bacteriuria. After a day of therapy with meropenem, her therapy was streamlined based on culture results to ceftriaxone. She continued to improve, her kidney function remained stable, and she was prescribed oral ciprofloxacin to complete a 14-day total course of antibiotics. This case is the first reported instance of *K. variicola* bacteremia associated with pyelonephritis in a renal transplant recipient. Hospitalization with acute pyelonephritis within the first year following kidney transplant is common and is associated with increased risk of graft loss and mortality. However, *K. variicola* is not a commonly known organism to cause this infection. Despite the risk of allograft failure in this circumstance, this patient was successfully treated with a 14-day course of antibiotic therapy.

## 1. Introduction

*Klebsiella variicola* (*K. variicola*) is a newly described Gram-negative *bacillus* included in the *Klebsiella pneumoniae* (*K. pneumoniae*) complex [1]. It was first identified in 2004 using DNA-DNA hybridization [2]. Members of the complex share many biochemical characteristics, making it challenging to differentiate them by routine biochemical methods in the laboratory [1]. *K. variicola* was originally thought to be an environmental pathogen that affected animals and plants [1]. However, recent studies have shown that *K. variicola* can cause a variety of diseases in humans, including meningitis [3] and bacteremia [4]. Blood stream infections with *K. variicola* were associated with a higher mortality rate than those with *K. pneumoniae* [5]. Here, we describe a case of *K. variicola* bacteremia in a patient 2 months after a renal transplant. Written consent was obtained from the patient to present this case.

## 2. Case Presentation

A 65-year-old woman with a longstanding history of end-stage renal disease secondary to polycystic kidney disease underwent renal transplantation from a deceased donor. The patient had good immediate allograft function with a creatinine of 130 *μ*mol/L on discharge. During admission posttransplant, she developed asymptomatic bacteriuria with extended-spectrum beta-lactamase- (ESBL-) producing *Escherichia coli* which was treated for 14 days with meropenem followed by ertapenem given her recent renal transplant. She received induction immunosuppression with basiliximab and intravenous methylprednisolone. She was discharged with a maintenance immunosuppressive regimen consisting of tacrolimus, mycophenolate, and prednisone.

Approximately 2 months posttransplant, the patient developed fever measuring 38.4°C and left lower quadrant abdominal pain over the renal allograft. Upon presentation to the emergency department, her blood pressure was 131/64 mmHg and her heart rate was 63 bpm. Her white blood cell count on admission was 11.7 × 10^9^ cells per litre and was predominantly neutrophils. Her creatinine was 134 *μ*mol/L which was essentially unchanged from her baseline. She endorsed some nausea and mild left-sided flank pain. She did not report any dysuria, gross hematuria, or increased urinary frequency. Her physical examination was significant for left lower quadrant abdominal tenderness. She was initially treated empirically with meropenem 500 mg IV q 8 h, given her history of ESBL-producing *E. coli* bacteriuria. Her fever resolved shortly after starting antibiotics. An abdominal CT scan showed inflammatory changes around the transplanted kidney that were suggestive of an infection rather than allograft rejection.

Her initial urine culture grew <10 × 10^6^ CFU/L of mixed enteric flora, but her imaging and clinical presentation were most in keeping with a urinary source. The blood culture signaled positive in 1 bottle after 16 hours. The initial gram stain showed Gram-negative bacilli resembling coliforms, and later, the isolate was identified as *K. variicola* by matrix-assisted laser desorption/ionization-time of flight (MALDI-TOF) mass spectrometry (Bruker Daltonics; Germany version 4.1 DB-7854). After a day of treatment with meropenem, her therapy was narrowed based on culture results to ceftriaxone 2 g IV daily. She continued to be afebrile, her white blood cell count normalized, and her pain gradually improved following 2 days of ceftriaxone. She was subsequently switched to ciprofloxacin 500 mg orally twice daily and discharged from the hospital. Her creatinine and GFR remained stable throughout her admission. She continued this treatment to complete a 14-day total course of antibiotics.

## 3. Discussion

This case is unique as it is the first reported instance of *K. variicola* bacteremia associated with pyelonephritis in a renal transplant recipient. One of the largest case series of *K. variicola* bacteremia, consisting of 34 patients, found none in previous organ transplant recipients [5]. Hospitalization with acute pyelonephritis within the first year following kidney transplant is common and is associated with increased risk of graft loss and mortality [6]. However, the most common organisms in acute pyelonephritis following transplant are *E. coli*, *Pseudomonas* species, and enterococci [7]. The peer-reviewed literature on *K. variicola* bacteremia was reviewed, and relevant available clinical patient cases are summarized in Table 1. The aggregate average age of all adult cases was 71 years with 70% being male. A range of organ involvement has been described in the literature including sinus, biliary, and urinary sources. The most common antibiotics used were cephalosporins and carbapenems. Previous comparative studies of different *Klebsiella* species have suggested that *K. variicola* is more prevalent in individuals aged greater than 80 years, whereas the patient in this case was significantly younger at 65 years of age [8]. A similar case was described in a patient who developed *K. variicola* bacteremia in the setting of ruptured appendicitis [9]. However, our case represents a higher-risk scenario given the lack of immediate source control and an at-risk allograft. Despite the risk of allograft failure in this circumstance, this patient was successfully treated with a 14-day antibiotic course with sequential use of meropenem, ceftriaxone, and ciprofloxacin. Four months after her initial presentation, the patient's kidney allograft is still functioning well with a creatinine of 95 *μ*mol/L.

Previous studies have highlighted the importance of differentiating *Klebsiella* species, as a fatal case of sepsis was described in a patient who had an isolate mistakenly thought to be *K. pneumoniae* but later found to be *K. variicola* [10]. The patient developed disseminated intravascular coagulation and multiorgan failure after 8 days despite being on meropenem [10]. *K. variicola* bacteremia is associated with higher mortality than bacteremia with *K. pneumoniae* [5]. Recent advancements in PCR-based technologies have improved the ability to differentiate these species and have found that 56% isolates of *K. variicola* tested were multidrug resistant [13].

In conclusion, bacteremia caused by *K. variicola* can carry significant mortality, and this species is often misidentified as *K. pneumoniae*. Here, we describe a case of acute pyelonephritis and bacteremia 2 months following renal transplant where accurate and prompt identification of *K. variicola* as the causative organism resulted in a favourable clinical outcome with preservation of allograft function.

## Figures and Tables

**Table 1:** Review of literature on *Klebsiella variicola*^a^ bacteremia.

Citation	Cohort size	Patient characteristics	Infection characteristics	Treatment^b^	Outcome
Ravin and Gandhi [9]	1	57-year-old woman with the history of hypothyroidism and breast cancer.	Necrotizing appendicitis leading to bacteremia. Initial blood cultures growing *K. variicola* and *Streptococcus* species.	Meropenem for 3 days and then 2 weeks of ceftriaxone 2 g IV.	Full recovery

Seki et al. [10]	1	67-year-old women with maxillary sinus cancer.	Sinus infection leading to bacteremia after course of chemotherapy. PCR analysis based on blood culture showed *K. variicola*.	Meropenem 3 g daily and vancomycin 2 g daily.	Developed disseminated intravascular coagulation and died from multiorgan failure after 8 days.

Fontana et al. [4]	1	72-year-old male with colorectal cancer and biliary stent following ascending cholangitis.	Biliary duct obstruction following stent placement leading to bacteremia. PCR analysis based on blood culture showed *K. variicola*.	Cefepime for 2 weeks.	Does not comment on case outcome.

Berry et al. [11]	1	27-year-old female with the history of systemic lupus erythematous complicated by lupus nephritis.	Admitted for hypoxic respiratory failure. Multiple blood cultures drawn throughout admission which were initially misidentified as *K. pneumoniae* but later found to be *K. variicola*.	Does not comment on specific antibiotics used.	Does not comment on case outcome.

Maatallah et al. [5]	34	Average age was 68 years with 77% male. Many (62%) had an underlying malignancy.	All had a bacteremia with urinary source being most common (35%). *K. variicola* was confirmed with PCR testing.	Cephalosporin, piperacillin-tazobactam, carbapenem, and/or ciprofloxacin^c^	Had a 29% 30-day mortality among *K. variicola* isolates, significantly higher than other *Klebsiella* species

Farzana et al. [12]	36	Neonates averaging 5.4 days old born at a Bangladesh hospital during the *K. variicola* outbreak.	Blood cultures were taken from all neonates presenting with clinical sepsis from October 2016 to March 2017.	All neonates received multiple antibiotics with overall usage being 62% ceftriaxone, 53% amikacin, 31% vancomycin, 16% gentamicin, 15% carbapenem, 9% azithromycin, 6% colistin, 6% metronidazole, and 3% ciprofloxacin.	Overall mortality of *K. variicola* bacteremia was 54.5% compared to a 33.3% sepsis mortality in an analogous non-*K. variicola* sepsis sample.

Imai et al. [8]	19	Average age was 81 years with 68% male. Many (37%) had an underlying malignancy.	All had bacteremia with the abdominal source being the most common (47%). *K. variicola* was confirmed with PCR testing.	Does not comment on specific antibiotics used.	Had a 11% 30-day mortality among *K. variicola* isolates not significantly different than other *Klebsiella* species

^a^*Klebsiella variicola* (*K. variicola).*^b^All dosing information that was available. ^c^Did not list treatments for *K. variicola *specifically among other Klebsiella species.

## Data Availability

The clinical patient data used to support the findings of this study are restricted by the University of British Columbia Clinical Research Ethics Board (UBC CREB) in order to protect patient privacy. Data are available from the corresponding author for researchers who meet the criteria for access to confidential data.
